# Predictive factors of psychiatric syndrome in patients with systemic lupus erythematosus

**DOI:** 10.3389/fimmu.2024.1323209

**Published:** 2024-03-21

**Authors:** Wenqi Geng, Shangzhu Zhang, Jinya Cao, Xia Hong, Yanping Duan, Yinan Jiang, Jing Wei

**Affiliations:** ^1^Department of Psychological Medicine, Chinese Academy of Medical Sciences & Peking Union Medical College, Peking Union Medical College Hospital, Beijing, China; ^2^Department of Rheumatology and Clinical Immunology, Chinese Academy of Medical Sciences & Peking Union Medical College, National Clinical Research Center for Dermatologic and Immunologic Diseases (NCRC-DID), Ministry of Science & Technology, State Key Laboratory of Complex Severe and Rare Diseases, Peking Union Medical College Hospital (PUMCH), Key Laboratory of Rheumatology and Clinical Immunology, Ministry of Education, Beijing, China

**Keywords:** systemic lupus erythematosus, neuropsychiatric systemic lupus erythematosus, mental disorders, biomarkers, referral consultation

## Abstract

**Introduction:**

Early detection of neuropsychiatric systemic lupus erythematosus (NPSLE) remains a challenge in clinical settings. Previous studies have found different autoantibodies as markers for NPSLE. This study aimed to describe the distribution of psychiatric syndromes in a group of patients with systemic lupus erythematosus (SLE) and to investigate the association between psychiatric syndromes and specific autoantibodies.

**Methods:**

This retrospective study was conducted at a single medical center in China. We reviewed medical records of hospitalized patients with SLE who were consulted by psychiatrists due to potential mental disorders. Results of serum autoantibodies and general laboratory tests were collected. The correlation between clinical variables was examined. Binary logistic regression analyses were used to determine factors related to NPSLE and different psychiatric diagnoses.

**Results:**

Among the 171 psychiatric manifestations in 160 patients, 141 (82.4%) were attributed to SLE. Acute confusional state (ACS) had the highest prevalence (57.4%). Anti-cardiolipin (ACL) antibody (*X*^2 = ^142.261, p < 0.001) and anti-β2 glycoprotein I (-β2GP1) antibody (*X*^2 = ^139.818, p < 0.001) varied significantly between groups, with the highest positive rate found in patients with mood disorders (27.3% and 18.2%). SLE disease activity index – 2000 (SLEDAI-2K) score excluding item ACS and item psychosis was a predictor of NPSLE (OR 1.172 [95% CI 1.105 - 1.243]).

**Conclusions:**

Disease activity reflected by SLEDAI-2K score is a predictor for NPSLE. Antiphospholipid antibodies are associated with mood disorders in SLE. Further separate investigation of neuropsychiatric disorders is needed in order to better comprehend NPSLE’s pathological mechanism.

## 1 Introduction

Systemic lupus erythematosus (SLE) is a systemic autoimmune disease that affects multiple organs or systems, characterized by excessive production of pathogenic autoantibodies (autoabs) against a range of autoantigens (1). Neuropsychiatric lupus (NPSLE) refers to SLE involving the nervous system, including 19 clinical syndromes such as epilepsy, psychosis, and acute confusional state, which are divided into two categories: central nervous system involvement and peripheral nervous system involvement (1). Due to the variety of clinical manifestations from mild to severe, early detection of NPSLE remains a challenge in clinical settings and frequently necessitates multidisciplinary collaboration (2, 3). As a sign of critical illness, NPSLE also predicts an increase in disability and mortality (2, 4). Hence, it is important to identify the risk of neuropsychiatric (NP) involvement in the early stages of the disease in order to achieve an optimal outcome.

The attribution of neuropsychiatric manifestations has been a major challenge in clinical settings, possibly due to the unelucidated etiopathogenesis of NPSLE. This complexity is also mirrored in the study outcomes, as the prevalence of NPSLE drastically span from 14% to 95% (5). Based on the American College of Rheumatology (ACR) Nomenclature of 19 NPSLE syndromes (6), Ainiala et al. distinguished between minor and major manifestations (7). Later attribution models also took into account chronological association of NP symptoms and SLE, in addition to confounding factors, such as the attribution model proposed by the Italian Study Group (8). Until this day, clinical diagnosis remains the gold standard for NPSLE.

Psychiatric syndromes in SLE include acute confusional state (ACS), anxiety disorders, cognitive dysfunction (CD), mood disorders, and psychosis, among which ACS and psychosis are considered of the most diagnostic or “organic” significance of NPSLE (6, 9), or major manifestations by Ainiala et al. (7). Anxiety disorders, mood disorders, and CD are common in patients with SLE, but their contributing factors are more complex. For instance, anxiety disorders and mood disorders may be related to psychosocial factors, while CD may be secondary to cerebrovascular disease (2, 10).

Vasculopathy, autoabs-mediated tissue and neuronal damage, inflammatory mediators, blood-brain barrier (BBB) dysfunction, and others are some of the suggested pathogenetic mechanisms for NPSLE (10, 11). Ho et al. proposed that autoabs such as anti-ribosomal P protein (-ribP) antibodies (abs), and anti-phospholipid (APL) abs may point to more “organic” causes, while patients with negative autoabs, and no positive results from head MRI and EEG results, point to “functional” causes. They also found that among 19 NP syndromes, the positivity of APL abs (serum anti-cardiolipin (ACL) abs, lupus anticoagulants (LA), anti-β2GP1) and anti-ribP abs were specifically significantly associated with the manifestations of mood disorder, psychosis, ACS, and CD (12). NP involvement might be aided by serum anti-Smith (-Sm) abs permeating BBB in patients with NPSLE (13, 14). Similar to the widely varying prevalence of NPSLE, biomarkers including autoabs varied from study to study, possibly as a result of different diagnostic criteria for NPSLE (15, 16).

The purpose of this study was to describe the distribution of psychiatric syndromes in patients with SLE and to investigate the association between psychiatric syndromes and specific autoabs and general laboratory results.

## 2 Methods

### 2.1 Subjects

We conducted this single-center retrospective cross-sectional study at Peking Union Medical College Hospital (PUMCH), a tertiary general hospital in Beijing, China. A review of the medical records of patients hospitalized at PUMCH between April 2013 and July 2020 was conducted by combining the efforts of both the Departments of Rheumatology and Clinical Immunology and Psychological Medicine.

The inclusion criteria of this study were: (a) Diagnosed as SLE by treating rheumatologists according to the 2012 Systemic Lupus Collaborating Clinics classification criteria for SLE (17); (b) Received psychiatric consultations due to potential mental disorders. Exclusion criteria were: (a) Comorbidity of other rheumatic diseases; (b) Incomplete information on psychiatric consultations or diagnoses. Psychiatric diagnoses were established through a full consideration of history, findings in mental status examination, and investigations. The diagnostic criteria for psychiatric syndromes in SLE, including ACS, psychosis, anxiety disorder, and mood disorder, were established upon the integration of the International Classification of Diseases Tenth Edition (ICD-10) (18) and the 1999 ACR NPSLE nomenclature (6). CD in NPSLE typically necessitates neuropsychological tests, such as the ACR neuropsychological battery for SLE. The standard procedure of mental status examination executed by consulting psychiatrists at PUMCH include general assessment of cognitive function, normally including tests of attention, memory, executive function and motor performance, verbal function and language skills, and concept formation and reasoning. Should the patient demonstrate dysfunction in a single domain, it is classified as mild CD. Should the patient exhibit cognitive decline in 2-3 domains, it is recognized as moderate CD. If the patient demonstrates impairments in 4-5 domains, it equates to severe CD. In this study, we defined CD related to SLE as the subject exhibiting impairment in at least two of the five cognitive domains.

The attribution of each patient’s psychiatric syndromes was determined by the attending rheumatologist, viewed as the gold standard. The primary basis for diagnosis and differential diagnosis is ACR Nomenclature for NPSLE (6) and EULAR recommendations for the management of SLE with neuropsychiatric manifestations (19). The information typically considered encompasses historical recollection, characteristics of symptoms, laboratory results, autoimmune antibodies, radiological and EEG observations, confounding factors that necessitate exclusion, and opinion from psychiatric consultation.

The study was conducted in accordance with the Helsinki Declaration of the World Medical Association. The design of the study was reviewed and approved by the Ethics Committee of PUMCH, and informed consent was waived due to observational/non-interventional design.

### 2.2 Data collection

General demographic information, medical features such as SLE disease activity index – 2000 (SLEDAI-2K) scores (20), psychiatric diagnoses, and usage of glucocorticoids (GC) and immunosuppressants (IS) were collected from medical records. Results of the following tests were also collected: complete hemogram, C3 and C4 levels, serum immunoglobulin (Ig), cerebrospinal fluid (CSF) analyses, anti-double stranded DNA (-dsDNA) abs, anti-Sm abs, anti-ribP abs, and the APL abs (LA, anti-β2 glycoprotein I (-β2GP1) abs, ACL abs). All abs were tested by enzyme-linked immunosorbent assay, and range of values to be considered negative were shown in **Supplementary Material 1**. Results of head MRI were also collected.

### 2.3 Statistical analyses

All statistical analyses were performed using IBM SPSS Statistics 21.0.0.0. (IBM Corp., Armonk, NY, USA). Categorical variables are described as frequencies (percentages). Quantitative variables are described as mean ± standard deviation or median (interquartile range [IQR]) based on the normality of distribution. Fisher’s 1-way ANOVA and Bonferroni correction or the Kruskal–Wallis test were used for among-group comparisons of continuous normally and non-normally distributed variables, respectively. The Chi-square test or Fisher’s exact test was used for among-group comparisons of categorical variables. The correlation between clinical variables and psychiatric diagnoses was examined using Spearman’s correlation test. Subsequently, binary logistic regression analyses were used to determine factors related to NPSLE and different psychiatric diagnoses, with age and gender incorporated into the model. A two-tailed p value < 0.05 was considered statistically significant.

## 3 Results

A total of 160 patients with SLE were included. Demographic information, clinical characteristics, and laboratory data of participants are summarized in **Table 1**; **Supplementary Material 2**. There were 145 women (90.6%), with a median age of 28 (IQR 23 - 39), and a median course of disease of 1.5 years (IQR 0.3 - 6). Seventy-five percentage of patients manifested multi-organ or -system involvement. The mean SLEDAI-2K score was 20.2 (SD 8.2). One hundred and forty-eight patients (92.5%) were diagnosed with at least one psychiatric condition. Of the 171 psychiatric manifestations, 141 (82.4%) were attributed to SLE. The highest to lowest prevalence was observed in ACS (81, 57.4%), CD (35, 24.8%), psychosis (14, 9.9%), and mood disorder (11, 7.8%), respectively.

**Table 1:** Intergroup comparisons of clinical features and autoantibodies.

	All patients N=160	ACS N=81	Psychosis N=14	Mood Disorders N=11		Cognitive Dysfunction N=35	None N=12	F/χ^2^	p
				Depressive N=1	Bipolar N=10				
Female, n (%)	145 (90.6)	75 (92.6)	12 (85.7)	Female	8 (80.0)	32 (91.4)	11 (91.7)	2.281	0.809
Age, median (IQR)	28 (23, 39)	28 (24, 41)	29 (23, 33)	47	26 (22, 41)	28 (23, 43)	26 (21, 30)	0.993	0.424
Disease course/yrs	1.5 (0.3, 6)	0.5 (0.2, 4.5)	2.8 (0.8, 6.0)	9	6.0 (2.7, 9.3)	1.5 (0.3, 7)	1.8 (0.2, 5.8)	1.569	0.172
SLEDAI-2K, mean ± SD	20.2 ± 8.2	21.4 ± 7.3	18.3 ± 9.3	22	9.3 ± 4.2	14.9 ± 7.4	14.5 ± 8.2	**7.526**	**<0.001**
SLEDAI-2K excluding ACS and psychosis, mean ± SD	13.6 ± 7.5	13.8 ± 7.0	11.4 ± 10.8	22	9.3 ± 4.2	14.9 ± 7.4	14.5 ± 8.2	1.164	0.330
APS n (%)	7 (4.3)	3 (3.7)	1 (7.1)	0 (0)	0 (0)	3 (8.6)	0 (0)	2.735	0.741
anti-dsDNA ab positivity, n (%)	61 (53.0)	47 (58.0)	7 (50.0)	Negative	7 (70.0)	16 (45.7)	8 (66.7)	2.885	0.718
anti-Sm ab positivity, n (%)	42 (29.0)	20 (24.7)	5 (35.7)	Negative	6 (60.0)	11 (31.4)	1 (11.1)	7.656	0.176
anti-ribP ab positivity, n (%)	50 (34.0)	30 (37.0)	5 (35.7)	Negative	5 (50.0)	15 (42.9)	5 (55.6)	2.513	0.775
ACL positivity, n (%)	23 (15.8)	10 (12.3)	1/12 (8.3)	Positive	3 (30.0)	3 (8.6)	1 (10.0)	142.261	**<0.001**
anti-β2GP1 ab positivity, n (%)	21 (14.6)	6 (7.4)	1/12 (8.3)	Positive	2 (20.0)	2 (5.7)	0 (0.0)	139.818	**<0.001**
LA/seconds, median (IQR)	1.2 (1.0, 1.2)	1.0 (0.9, 1.1)	1.1 (1.0, 1.3)	/	1.1 (1.0, 1.2)	1.0 (0.9, 1.2)	1.1 (1.0,1.1)	0.879	0.479

ACS, acute confusional state. APS, antiphospholipid syndrome. anti-dsDNA, anti-double stranded DNA antibodies. anti-Sm, anti Smith antibodies. anti-ribP, anti-ribosomal P, protein antibodies. ACL, anti-cardiolipin antibodies. anti-β2GP1, anti-β2 glycoprotein I antibodies. LA, lupus anticoagulants.p values suggesting statistical significance (p < 0.05) was in bold font."/" stands for not applicable (N/A).

Compared between groups, there were no significant differences in sex ratio, age, age of onset, or course of disease. **Tables 1**, **2** depict that the mean SLEDAI-2K score demonstrated a significant variance amongst diagnostic groups (F = 7.526, p < 0.001). Removal of items pertaining to ACS and psychosis, revealed no significant differences in the remainder of the SLEDAI-2K score between groups (F = 1.164, P = 0.330). Patients presenting with bipolar disorder secondary to SLE displayed the lowest mean SLEDAI-2K score of 9.3 ± 4.2. Seven patients (4.3%) were diagnosed concurrently with antiphospholipid syndrome (APS). Three patients experienced ACS, three presented with CD, and one exhibited psychosis.

**Table 2:** SLEDAI-2K results.

Item n (%)	All patients N=160	ACS N=81	Psychosis N=14	Mood Disorders N=11		Cognitive Dysfunction N=35	None N=12
				Depressive N=1	Bipolar N=10		
Seizure	29 (18.1)	14 (17.3)	1 (7.1)	1	1 (10.0)	5 (14.3)	2 (16.7)
Psychosis	14 (8.8)	0 (0)	14 (100.0)	0	0 (0)	0 (0)	0 (0)
Organic brain syndrome	81 (50.6)	81 (100.0)	0 (0)	0	0 (0)	0 (0)	0 (0)
Visual disturbance	6 (3.8)	4 (4.9)	1 (7.1)	0	0 (0)	2 (5.7)	1 (8.3)
Cranial nerve disorder	2 (1.3)	2 (2.5)	0 (0)	0	0 (0)	1 (2.9)	0 (0)
Lupus headache	13 (8.1)	6 (7.4)	0 (0)	1	1 (10.0)	4 (11.4)	2 (16.7)
CVA	6 (3.8)	1 (1.2)	0 (0)	0	0 (0)	1 (2.9)	0 (0)
Vasculitis	11 (6.9)	6 (7.4)	1 (7.4)	0	0 (0)	3 (8.6)	1 (8.3)
Arthritis	28 (17.5)	14 (17.3)	3 (21.4)	0	0 (0)	5 (14.3)	2 (16.7)
Myositis	10 (6.3)	6 (7.4)	1 (7.4)	0	0 (0)	5 (14.3)	1 (8.3)
Urinary casts	7 (4.4)	3 (3.7)	0 (0)	0	0 (0)	3 (8.6)	1 (8.3)
Hematuria	39 (24.4)	23 (28.4)	2 (14.3)	0	2 (20.0)	10 (28.6)	3 (25.0)
Proteinuria	72 (45.0)	43 (53.1)	3 (21.4)	0	4 (40.0)	18 (51.4)	7 (58.3)
Pyuria	4 (2.5)	3 (3.7)	0 (0)	0	0 (0)	2 (5.7)	0 (0)
New rash	57 (35.6)	31 (38.3)	6 (42.9)	0	3 (30.0)	17 (48.6)	6 (50.0)
Alopecia	28 (17.5)	16 (19.8)	3 (21.4)	0	2 (20.0)	6 (17.1)	3 (25.0)
Mucosal ulcers	28 (17.5)	15 (18.5)	2 (14.3)	0	1 (10.0)	10 (28.6)	3 (25.0)
Pleurisy	32 (20.0)	17 (21.0)	1 (7.4)	1	2 (20.0)	6 (17.1)	2 (16.7)
Pericarditis	22 (13.8)	14 (17.3)	0 (0)	1	2 (20.0)	4 (11.4)	0 (0)
Low complement	105 (65.6)	57 (70.4)	10 (71.4)	1	6 (60.0)	26 (74.3)	8 (66.7)
Increased DNA binding	84 (52.5)	40 (49.4)	9 (64.3)	0	5 (50.0)	15 (42.9)	8 (66.7)
Fever	53 (33.1)	26 (32.1)	5 (35.7)	0	2 (20.0)	10 (28.6)	7 (58.3)
Thrombocytopenia	69 (43.1)	39 (48.1)	5 (35.7)	1	3 (30.0)	18 (51.4)	4 (33.3)
Leukopenia	64 (40.0)	30 (37.0)	4 (28.6)	1	4 (40.0)	20 (57.1)	7 (58.3)

ACS, acute confusional state. CVA, cerebrovascular accident.

All patients were prescribed GC as standardized therapy, and cortisol pulse therapy was implemented in 89 patients (55.6%). The most to least frequently administered IS were cyclophosphamide (66.3%), hydroxychloroquine (61.3%), mycophenolate mofetil (18.1%), tacrolimus (8.8%), cyclosporine A (4.4%), belimumab (1.9%), leflunomide (1.9%), and thalidomide (1.9%).

In relation to specific autoantibodies in various diagnostic cohorts, more patients carried positive ACL and anti-β2GP1 abs in the group of mood disorders (36.4% and 27.3). No notable differences in the positivity rates of anti-dsDNA abs, anti-Sm abs, and anti-ribP abs existed across groups. Additionally, there was no significant difference in hemoglobin, white blood cell count, platelet, plasma Igs, C3, C4, LA, and CSF analyses among diagnostic groups (**Supplementary Material 2**).

Of the 132 patients who underwent head MRI, abnormal results were noted in 85 patients (64.4%). These included 14 presenting with ischemic lesions, 37 with inflammatory lesions, 5 exhibiting both ischemic and inflammatory lesions, and 29 demonstrating other abnormalities. Notably, the findings of head MRI outcomes did not significantly differ across patients with disparate psychiatric diagnoses, as indicated in **Table 3**. Of the seven patients with secondary APS, four had ischemic lesions, two demonstrated both ischemic and inflammatory lesions, while the remaining patient did not undergo MRI.

**Table 3:** Intergroup comparisons of head MRI results.

MRI abnormality	All patients N=132	ACS N=55	Psychosis N=12	Mood Disorders N=10		Cognitive Dysfunction N=33	None N=10	χ^2^	p
				Depressive N=1	Bipolar N=9				
Ischemic	37 (28.0)	18 (32.7)	1 (8.3)	1	1 (11.1)	4 (12.1)	2 (20.0)	5.999	0.306
Inflammatory	14 (10.6)	6 (10.9)	5 (41.7)	/	3 (33.3)	6 (18.2)	4 (40.0)	1.507	0.912
Inflammatory + ischemic	5 (3.8)	3 (5.6)	0 (0)	/	0 (0)	2 (6.1)	0 (0)	1.961	0.854
Other	29 (22.0)	13 (23.6)	0 (0)	/	1 (11.1)	10 (30.3)	2 (20.0)	6.796	0.236
Normal	47 (35.6)	26 (47.3)	6 (50.0)	/	4 (44.4)	11 (33.3)	2 (20.0)	2.824	0.827

ACS, acute confusional state."/" stands for not applicable (N/A).

Correlation analysis revealed that bipolar disorder exhibited a weak correlation with the SLEDAI-2K score excluding the ACS and psychosis item (r=-0.167, p=0.039), course of disease (r=0.170, p=0.035), and anti-Sm ab (r=0.185, p=0.023). Psychosis demonstrated a weak correlation with hemoglobin (r=0.187, p=0.020). ACS and CD were not significantly correlated with any clinical features presented. The SLEDAI-2K score excluding the ACS and psychosis item demonstrated a moderate correlation with NPSLE (r=0.421, p<0.001). Binary logistic regression revealed that the SLEDAI-2K score excluding the ACS and psychosis item was a predictor of NPSLE (OR 1.172 [95% CI 1.105 - 1.243]). With the available data, we did not identify distinct indicators for each specific psychiatric syndrome.

## 4 Discussion

In this research, we analyzed the correlation between clinical variables and NPSLE, concentrating predominantly on psychiatric disorders. Identifying biomarkers associated with the manifestation of diverse syndromes may foster comprehension of the pathogenesis of NPSLE. Psychiatric disorders in SLE, as secondary psychiatric disorders, suggest involvement of the CNS. Comparable to primary mental disorders, whilst there may be common pathological mechanisms among diverse types of syndromes, such as those of NPSLE, it should likewise exist distinct pathological mechanisms, such as those exclusive to psychosis. Presently, a definitive “gold standard” is lacking, and previous endeavors to delineate biomarkers for NPSLE have yielded conflicting results (21). Differences in diagnostic criteria and the absence of standardized reporting may be two possible explanations (15, 22). For instance, from a psychiatric perspective, mood disorders are two types of mental disorders of diverse nature, as both depressive and bipolar disorders consist of several subtypes. Consequently, markedly heterogeneous conclusions are likely to emerge from various studies concerning mood disorders in SLE. Moreover, the exploration of the genesis of primary psychiatric disorders necessitates consideration of the influence of social and psychological factors. Regrettably, in the majority of research pertaining to NPSLE, inclusive of this study, psychometric evaluation data have been omitted or incomplete (5). This deficiency should be rectified in subsequent investigations.

Our study demonstrated that the SLEDAI-2K score exhibits distinctions in disease activity among patients with varied psychiatric syndromes. As ACS and mental disorders are constituents of the SLEDAI-2K score, the SLEDAI-2K global score of patients experiencing ACS or psychosis may surpass other patients who do not fulfill these two classifications. Following the exclusion of these two items, it was determined that there exists no notable disparity in disease activity amongst different diagnostic cohorts. Moreover, there weren’t noteworthy intergroup discrepancies in the majority of investigations, potentially due to the minimal sample size within certain subgroups. The correlation between the SLEDAI-2K score and NPSLE is predictable, as NPSLE is often a sign of critical illness (4). Upon our attempt to remove the ACS and psychosis items, it was evident that the remaining SLEDAI-2K score retained its capability of predicting NPSLE, providing evidence for a strong correlation between disease activity and the manifestation of psychiatric symptoms in SLE patients.

Thus far, research has focused on the association of autoabs with NP conditions in SLE (4). Anti-dsDNA ab, one of the hallmark markers of SLE, is associated with overall disease activity and renal involvement. However, its correlation with neuropsychiatric manifestations was not remarkable (23). Research on anti-Sm ab, another diagnostic marker of SLE, revealed a significant increase in anti-Sm ab in patients with ACS compared to those with focal NPSLE and the fact that it plays a significant role in disrupting BBB in NPSLE (13, 14). Despite the controversy surrounding the conclusion, anti-ribP ab has become known as a highly specific biomarker for the diagnosis of SLE and is frequently associated with NPSLE (24). The presence of positive anti-ribP antibodies at baseline was associated with a greater proportion of neurological involvement (adjusted HR = 3.8, 95% CI 2.7-57) and cumulative neuropsychiatric damage during follow-up in our colleagues’ recent study, which focused on NPSLE (25). In a recent cross-sectional study by Chessa et al., anti-ribP serum level was independently associated with depressive symptoms (26). In our present study, however, the aforementioned abs did not differ significantly between psychiatric diagnostic groups. Hanly et al. discovered that impairment in BBB function is an important contributor to cognitive dysfunction, regardless of circulating SLE-related autoantibodies (27). The correlation between autoantibodies and NPSLE may comprise other mediating factors, necessitating future investigation.

The presence of APL abs is commonly associated with thrombosis (28). In NPSLE, APL ab-mediated thrombosis is considered the etiology of cerebrovascular disease and a possible mechanism for CD (21, 29). Owing primarily to the limited number of patients exhibiting APS in our research, no discernible difference was detected in the prevalence of APS across distinct psychiatric diagnoses. Nonetheless, we did discover that ACL and anti-β2GP1 ab were more frequently observed in patients with mood disorders, a finding consistent with some prior studies. In a previous meta-analysis, anti-β2GP1 ab was found to be significantly associated with mood disorders (OR=6.27 [95% CI, 1.22-32.12]) (12). According to findings from a study focusing on perinatal women with APS, even after pregnancy, women with pure obstetric APS are at increased risk of venous and arterial thrombosis over time, and they also appear to develop more mood disorders (30). Early theoretical models postulated that mood disorders are due to malfunctioning of several neurotransmitters, while recent research has uncovered more neurobiological mechanisms, including vascular dysfunction (31, 32). Phenomena of cerebral hypoperfusion were observed in both depressive and bipolar disorders (33, 34). In order to better elucidate the pathological mechanism of APL abs in mood disorders in SLE, it is important to investigate depressive and bipolar disorders separately in future studies with larger sample sizes.

The main strength of our study lies in that we focused on psychiatric syndrome of patients with SLE, which maximizes the accuracy of diagnoses. Meanwhile, several limitations should not be overlooked. First, as a result of the small sample size of each diagnostic group, certain intergroup differences in autoabs and clinical features may not be fully reflected. Second, the study was conducted at a single medical center in Beijing, China, which may limit the generalizability of the results. Third, due to the retrospective design, data on some clinical confounding factors may have been missed. Validation of these findings requires large-scale prospective multi-center studies, as well as more in-depth research to elucidate the pathological mechanisms underlying NPSLE.

## 5 Conclusions

Disease activity reflected by SLEDAI-2K score is a predictor for NPSLE. Antiphospholipid antibodies are associated with mood disorders in SLE. A detailed exploration of neuropsychiatric disorders, ideally inclusive of psychosocial evaluations, is imperative to elaborate on the pathogenic mechanism of NPSLE.

## Data availability statement

The raw data supporting the conclusions of this article will be made available by the authors, without undue reservation.

## Ethics statement

The studies involving humans were approved by Ethics committee of Peking Union Medical College Hospital. The studies were conducted in accordance with the local legislation and institutional requirements. The ethics committee/institutional review board waived the requirement of written informed consent for participation from the participants or the participants’ legal guardians/next of kin because of observational/non-interventional design.

## Author contributions

WG: Writing – review & editing, Writing – original draft, Data curation. SZ: Writing – review & editing, Writing – original draft, Methodology. JC: Writing – review & editing, Writing – original draft, Validation, Supervision, Conceptualization. XH: Writing – review & editing, Supervision. YD: Writing – review & editing. YJ: Writing – review & editing, Project administration, Methodology. JW: Writing – review & editing, Supervision, Funding acquisition.
