# Supplementary material for: Predictive factors of psychiatric syndrome in patients with systemic lupus erythematosus
Source: Front Immunol. 2024 Mar 21;15:1323209. doi: 10.3389/fimmu.2024.1323209 (PMC10996363; doi:10.3389/fimmu.2024.1323209)
Supplement: Supplementary file 1 [file Table_1.docx]

Supplementary material 1. Range of values of autoantibodies

|  | Negative | Suspicious | Positive |
| --- | --- | --- | --- |
| ACL-IgG  (GPLU/ml) | <8.0 | 8.0-<12.0 | ≥12.0 |
| ACL-IgM  (MPLU/ml) | <8.0 | 8.0-<12.0 | ≥12.0 |
| ACL-IgA  (APLU/ml) | <8.0 | 8.0-<12.0 | ≥12.0 |
| β2GP1-IgG  (AU/ml) | <16.0 | 16.0-<24.0 | ≥24.0 |
| β2GP1-IgM  (AU/ml) | <16.0 | 16.0-<24.0 | ≥24.0 |
| β2GP1-IgA  (AU/ml) | <16.0 | 16.0-<24.0 | ≥24.0 |
| LA |  |  | >1.20 |
| Sm  (AI) | <10 |  |  |
| ribP  (AI) | <10 |  |  |
| dsDNA-IgG  (IU/ml) | <24.0 | 24.0-<36.0 | ≥36.0 |

ACL, anti-cardiolipin. β2GP1, anti-β2 glycoprotein I. LA, lupus anticoagulants. Sm, Smith. ribP, anti-ribosomal P protein.
