# Supplementary material for: Predictive factors of psychiatric syndrome in patients with systemic lupus erythematosus
Source: Front Immunol. 2024 Mar 21;15:1323209. doi: 10.3389/fimmu.2024.1323209 (PMC10996363; doi:10.3389/fimmu.2024.1323209)
Supplement: Supplementary file 2 [file Table_2.docx]

Supplementary material 2. Additional results of intergroup comparisons of clinical features

|  | All patients  N=160 | ACS  N=81 | Cognitive Dysfunction N=35 | Psychosis  N=14 | Depressive Disorders N=1 | Bipolar Disorder N=10 | None  N=12 | F | P |
| --- | --- | --- | --- | --- | --- | --- | --- | --- | --- |
| Age at SLE diagnosis, median(IQR) | 25(17, 35) | 27(20, 38) | 26(17, 37) | 26(18, 32) | 38 | 23(16, 34) | 22(16, 25) | 1.415 | 0.222 |
| IgG, median(IQR) | 11.5(9.1，18.2) | 13.3(9.5, 18.3) | 12.4(10.0, 20.8) | 13.2(9.3, 17.3) | / | 10.6(7.5, 17.7) | 11.2(9.6,18.6) | 0.492 | 0.741 |
| IgM, median(IQR) | 0.82(0.54，1.27) | 0.87(0.54, 1.27) | 0.87(0.54, 1.64) | 0.75(0.57, 1.01) | / | 0.60(0.52, 0.74) | 0.60(0.46,1.60) | 0.740 | 0.566 |
| IgA, median(IQR) | 2.0(1.4, 2.6) | 1.9(1.4, 2.5) | 2.2(1.6, 3.2) | / | 1.3(1.0, 2.5) | 2.1(1.6, 2.6) | 2.4(2.1,3.6) | 1.733 | 0.146 |
| C3, median(IQR) | 0.52(0.35, 0.76) | 0.49(0.36, 0.69) | 0.46(0.29, 0.73) | 0.53(0.32, 0.83) | / | 0.62(0.27, 0.81) | 0.40(0.26, 0.91) | 0.304 | 0.875 |
| C4, median(IQR) | 0.09(0.04, 0.13) | 0.08(0.04, 0.12) | 0.07(0.02, 0.13) | 0.09(0.03, 0.14) | / | 0.10(0.01, 0.16) | 0.08(0.03, 0.12) | 0.015 | 1.000 |
| HGB, mean±SD | 100.4±25.1 | 95.7±23.8 | 93.9±20.5 | 111.6±22.6 | 112 | 96.4±18.7 | 110.1±24.4 | 2.169 | 0.061 |
| PLT, median(IQR) | 153(104, 226) | 143(95, 214) | 143(92, 193) | 156(124, 271) | 104 | 196(85, 264) | 135(107, 207) | 0.868 | 0.505 |
| WBC, median(IQR) | 5.8(3.7,9.6) | 6.1(4.0, 10.1) | 5.3(3.1, 8.3) | 5.3(3.5, 6.5) | 13.41 | 6.3(3.1, 9.4) | 4.3(2.7,9.0) | 0.290 | 0.918 |
| CSF-Pro, median(IQR) | 0.35(0.26，0.53) | 0.36(0.26, 0.55) | 0.44(0.31, 0.63) | 0.36(0.22, 0.50) | 0.26 | 0.26(0.18, 0.83) | 0.31(0.22,0.45) | 0.757 | 0.583 |
| CSF-Glu, mean±SD | 3.3±0.7 | 3.3±0.7 | 3.2±0.8 | 3.4±0.9 | 5.0 | 3.0±0.5 | 3.3±0.7 | 1.461 | 0.207 |
| CSF-Cl, mean±SD | 123.7±4.9 | 123.7±5.6 | 123.5±4.5 | 123.1±2.7 | 123 | 125.6±4.8 | 126.3±3.4 | 0.770 | 0.573 |
| CSF-Cell count, median(IQR) | 2.0(0，55.0) | 2.9(0, 106.0) | 2.0(0, 53.0) | 0(0, 5.0) | 56 | 4.0(1.0, 52.0) | 29.0(1.0,375.0) | 2.367 | 0.043 |
| CSF-WBC, median(IQR) | 0(0，2.0) | 0(0, 2.0) | 0(0, 2.0) | 0(0, 2.0) | 8 | 0(0, 4.0) | 3.0(0.0,12.5) | 0.413 | 0.839 |

ACS, acute confusional state. IgG, immunoglobin G. IgM, immunoglobin M. IgA, immunoglobin A. HGB, hemoglobin. PLT, platelet. WBC, white blood cell. CSF, cerebral spinal fluid. CSF-Pro, CSF protein. CSF-Glu, CSF glucose. CSF-Cl, CSF chloride.
